# Effects of NatureKnit™, a Blend of Fruit and Vegetable Fibers Rich in Naturally Occurring Bound Polyphenols, on the Metabolic Activity and Community Composition of the Human Gut Microbiome Using the M-SHIME^®^ Gastrointestinal Model

**DOI:** 10.3390/microorganisms13030613

**Published:** 2025-03-07

**Authors:** Marlies Govaert, Cindy Duysburgh, Brendan Kesler, Massimo Marzorati

**Affiliations:** 1 ProDigest, Technologiepark 82, 9052 Zwijnaarde, Belgium; marlies.govaert@prodigest.eu (M.G.); cindy.duysburgh@prodigest.eu (C.D.); 2 VDF FutureCeuticals, Inc., 2692 N. State Rt. 1-17, Momence, IL 60954, USA; brendan.kesler@futureceuticals.com; 3 Center for Microbial Ecology and Technology (CMET), Faculty of Bioscience Engineering, Ghent University, Coupure Links 653, 9000 Gent, Belgium

**Keywords:** fiber, gut microbiome, inulin, Mucosal Simulator of the Human Intestinal Microbial Ecosystem (M-SHIME^®^), polyphenol, bound polyphenols, prebiotic, psyllium, short-chain fatty acid (SCFA)

## Abstract

This study evaluated the impact of a proprietary blend of fruit and vegetable fibers rich in naturally occurring bound polyphenols (commercially marketed as NatureKnit^TM^), compared to purified fibers (inulin and psyllium), on the human gut microbiome using the validated M-SHIME^®^ gastrointestinal model. A short-term single-stage colonic M-SHIME^®^ experiment (with fecal inoculum from three healthy human donors) was used to evaluate the test products compared to a negative control. Samples were assessed for pH, gas pressure, short-chain fatty acid (SCFA) production, lactate, and ammonium from 0 h to 48 h. Microbial community composition was assessed at 0 h (negative control only), 24 h, and 48 h (lumen) or 48 h (mucosal). All test products were fermented well in the colon as demonstrated by decreases in pH and increases in gas pressure over time; these changes occurred faster with the purified fibers, whereas NatureKnit™ demonstrated slow, steady changes, potentially indicating a gentler fermentation process. SCFA production significantly increased over the course of the 48 h experiment with all test products versus negative control. SCFA production was significantly greater with NatureKnit™ versus the purified fibers. Shifts in the microbial community composition were observed with all test products versus negative control. At the conclusion of the 48 h experiment, the absolute bacterial abundance and the richness of observed bacterial taxa in the lumen compartment was significantly greater with NatureKnit™ compared with inulin, psyllium, and negative control. Overall, NatureKnit™ demonstrated greater or similar prebiotic effects on study measures compared with established prebiotic fibers.

## 1. Introduction

Dietary fiber is considered a component of public health concern for the general US population, as underconsumption is associated with health concerns [1]. The vast majority of people in the US have an inadequate intake of dietary fiber, with over 90% of women and 97% of men failing to meet the US Adequate Intake level. Underconsumption of dietary fiber can lead to the onset or progression of diet-related chronic conditions or diseases such as cardiovascular disease, obesity, and type 2 diabetes. The failure to meet guideline-recommended fiber intake is mainly driven by the fact that a high percentage of the US population does not consume the recommended daily intake of whole grains (nearly 100%), vegetables (nearly 90%), and fruit (nearly 80%) [1,2]. The lack of sufficient dietary fiber intake is not limited to the US. It is also reported in Europe [3], South America [4,5], Asia [6,7], South Africa [8], and Australia [9], indicating that the fiber gap is a global health issue.

Polyphenols are compounds found in plants (e.g., fruits, vegetables, cereals, tea, and wine). A high dietary intake of these compounds is associated with a reduced risk of several diseases, including cardiovascular disease, cancer, and neurodegenerative disorders [10]. A significant portion of dietary polyphenols are non-extractable polyphenols that are bonded with dietary fiber [11,12,13]. Unlike extractable polyphenols, non-extractable polyphenols cannot be absorbed in the small intestine [12,13,14]. Instead, these fiber-bound non-extractable polyphenols travel to the large intestine region where they are catabolized by the colonic microbiota [12,13,15]. The resulting metabolites promote homeostasis in the colon, have antioxidant activity, and promote balance of the gut microbiota [12,13,16,17]. Consequently, insufficient fiber consumption may also result in a reduced intake of health-promoting polyphenols.

Prebiotics are defined as “a substrate that is selectively used by host microorganisms conferring a health benefit” [18]. Prebiotics include non-digestible fibers that are resistant to digestion and absorption in the small intestine but are partially or completely fermented by bacteria in the colon through a process of saccharolytic fermentation [19]. An important metabolic byproduct of this type of fermentation is health-promoting short-chain fatty acids (SCFAs) [19,20]. Prebiotics promote a shift in the gut microbiota, supporting the enhanced growth of saccharolytic bacteria (e.g., *Bifidobacterium*) and resulting in elevated levels of SCFAs [21,22]. This shift may lead to a decrease in proteolytic bacteria and a consequent reduction of branched SCFAs, ammonium, and other toxic byproducts of proteolytic fermentation.

Fruits and vegetables are natural food sources of prebiotics [23]. In fact, much of the research elucidating the health benefits of fiber has come from studies of dietary fiber consumed as a part of food rather than from evaluations of purified prebiotic fiber supplements [24]. Inulin and psyllium are examples of two widely commercialized purified fibers that meet the prebiotic definition [25,26,27,28,29,30]. These purified fibers, however, do not typically have the vitamins, minerals, and other nutrients associated with fiber-rich fruits and vegetables [23]. Purified fibers may also be void of polyphenols, which have recently emerged in research as potential prebiotics [23]. Finally, purified fibers may cause gastrointestinal distress and bloating, particularly if they are rapidly fermented [31,32]. 

The purpose of our research was to analyze how a blend of fruit and vegetable fibers, rich in naturally occurring bound polyphenols, would compare to common purified fibers in (1) fermentation profile and (2) prebiotic effect. While it would be ideal to perform in-depth investigations of the effects of prebiotic fibers and polyphenols in clinical studies, they are limited in that only in and output data are obtained. This “black-box effect” prevents detailed study of the microbial metabolic activity and community composition along the gastrointestinal tract (GIT) directly in humans. Animal studies can act as a surrogate, but they are also limited as the background colonic microbiota, anatomy, and physiology of the GIT (pH, bile salt levels, retention time, and temperature) differ considerably between humans and animals [33,34]. To facilitate a more detailed analysis of microbial metabolism and community composition along the GIT, in vitro models that accurately simulate the physiological conditions of the entire human GIT and allow for sampling from each region at multiple timepoints have been developed [35,36,37]. The Mucosal Simulator of the Human Intestinal Microbial Ecosystem (M-SHIME^®^) is a validated model of the human intestine that utilizes fecal samples from human donors [38]. This model has been shown to accurately mimic in vivo conditions [38]. Models such as the M-SHIME^®^ that use microbiomes from human donors allow for ex vivo studies that aid in uncovering in-depth mechanical insights into the mode-of-action of prebiotics and other supplements, complementing clinical trial observations and providing a more complete understanding of their effects.

This study evaluated the impact of NatureKnit™, a blend of fruit and vegetable fibers rich in naturally occurring bound polyphenols, compared to two purified fibers, inulin and psyllium, on the human fecal microbiota metabolic activity and community composition in the colon. Short-term colonic simulations were therefore performed using the validated M-SHIME^®^ technology platform.

## 2. Materials and Methods

### 2.1. Test Products

The fruit and vegetable fiber blend (commercially known as NatureKnit^TM^) is comprised of a proprietary blend of apple fiber, carrot fiber, cranberry fiber, blueberry fiber, and whole spinach. Among other things, the blend contains at least 50% dietary fiber and naturally occurring fiber-bound polyphenols of 1 to 1.5% (Futureceuticals, Momence, IL, USA). Two common purified fibers, inulin and psyllium, were used for comparison in the short-term colonic simulations (specifications of the purified fibers are available upon request).

### 2.2. Short-Term Colonic Simulation

Fresh human fecal samples were collected from three healthy adults (male, *n* = 1; female, *n* = 2). Healthy donors were characterized as having a healthy body mass index (18.5 to 24.9), no diagnosed diseases which could result in a dysbiosed gut microbiota (e.g., inflammatory bowel disease, irritable bowel syndrome, Parkinson’s disease, diabetes), had not taken antibiotics in the four months prior to fecal sample collection, and aged between 20 and 45 years. Fecal materials were collected and used as approved by the Ethics Committee of the University Hospital Ghent (reference number ONZ-2022-0267).

A short-term single-stage colonic M-SHIME^®^ experiment was carried out to explore the microbial metabolic activity and community composition following the fermentation of the test products in a simulated colon model [39,40,41]. This model has been validated for representativeness of the human in vivo situation in multiple studies [35,42,43,44] and has been widely used to evaluate the effects of test products/conditions on the human gut microbiota [45,46,47,48,49]. At the beginning of the short-term colonic incubation, either no test product (negative control) or one of the test products (NatureKnit™, inulin, psyllium; fiber-matched so that each test condition received 1.667 g fiber/L) were added to 63 mL fresh carbohydrate-depleted medium representative for the colonic environment (nutritional medium PD01; ProDigest, Gent, Belgium). The amount of fiber used in the study was calculated to represent the physiological equivalent of 1 g fiber in a human. Fresh fecal inoculum from one of three healthy donors was then added (7 mL) to simulate a metabolically active colonic microbial community. The mucosal layer was simulated by inserting five mucus-coated carriers into each colonic reactor. The reactors were made anaerobic by flushing with nitrogen gas. Incubations were caried out for 48 h (37 °C with shaking [90 rpm]). All conditions were performed in triplicate to account for biological variation. 

Samples were collected at 0 h, 6 h, 24 h, and 48 h for assessment of pH, gas pressure, SCFA, lactate, and ammonium (fermentation profiling and microbial metabolic activity). Samples for assessment of the luminal microbial community composition were collected at 0 h (negative control only), 24 h, and 48 h. The mucosal microbial community composition was only assessed at 48 h.

### 2.3. Fermentation Profiling and Microbial Metabolic Activity

pH changes were measured using a Senseline F410 pH meter (ProSense, Oosterhout, The Netherlands). Gas pressure was measured using a hand-held pressure indicator (CPH6200; Wika, Echt, The Netherlands). The methods of De Weirdt et al. were used to measure SCFAs (acetate, propionate, and butyrate) and branched SCFAs [50]. An Enzytec™ kit was used to measure lactate concentrations according to the manufacturer’s instructions (R-Biopharm, Darmstadt, Germany). Ammonium levels were measured according to the method of Tzollas et al. [51].

### 2.4. DNA Extraction and 16S rRNA Sequencing

Total DNA was isolated as described by Duysburgh et al. [52]. 16S-targeted sequencing was accomplished using primers spanning two hypervariable regions (V3-V4) of the 16S rRNA gene (341F, 5′-CCTACGGGNGGCWGCAG-3′; 785R, 5′-GACTACHVGGGTATCTAAKCC-3′) [53,54]. Using a pair-end sequencing approach, sequencing of 2 × 250 bp resulted in 424 bp amplicons (LGC Genomics GmbH, Berlin, Germany). Fragments of this size are taxonomically more informative than smaller ones. The Schloss lab MiSeq SOP was used for read assembly and cleanup [54,55]. Briefly, mothur (v.1.44.3) was used to assemble reads into contigs, perform alignment-based quality filtering (alignment to the mothur-reconstructed SILVA SEED alignment, v138), remove chimeras (vsearch v2.13.3), assign taxonomy using a naïve Bayesian classifier [56] and SILVA NR v138_1, and cluster contigs into Operational Taxonomic Units (OTUs) at 97% sequence similarity. All sequences that could not be classified and those that were classified as Eukaryota, Archaea, chloroplasts, and mitochondria were removed. The most abundant sequence within an OTU was used as the representative.

### 2.5. Quantification of Total Bacterial Cells in the Lumen Samples

Flow cytometry was used to determine the total number of bacterial cells in the luminal samples. This allowed for the conversion of the metagenomics data from relative abundances to absolute abundances by multiplying relative abundances in a sample with the total cell count [57]. Samples were analyzed using a BD Accuri C6 Plus Flow Cytometer (BD Biosciences, Franklin Lakes, NJ, USA) using the high flow rate setting. A threshold level of 700 on the SYTO channel was used to separate bacterial cells from medium debris and signal noise.

### 2.6. Statistical Analysis

Differences in pH, gas pressure, SCFA, ammonium, and branched SCFA were determined over the course of the entire colonic incubation (i.e., between 0 h and 48 h incubation). For lactate, differences were determined over the course of the initial time interval (i.e., between 0 h and 6 h incubation). Statistically significant differences between different test conditions were determined using a paired student’s *t*-test (*p* < 0.05).

Alpha diversity was analyzed using three common indices: Shannon (species richness and evenness), Inverse Simpson (species richness and evenness, giving more weight to common or dominant species), and observed taxa (species richness).

## 3. Results

### 3.1. Fermentation Profile

**Figure 1:**
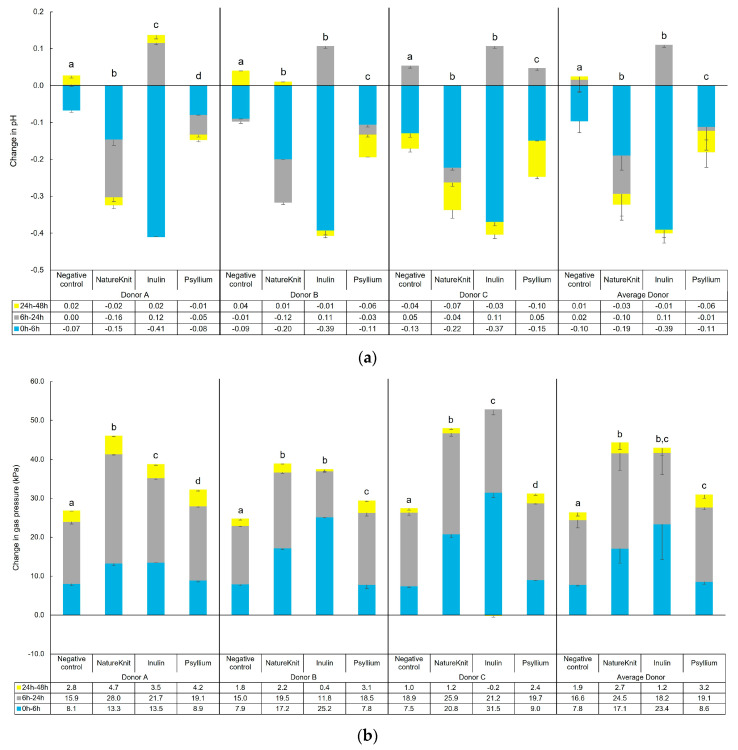
Changes in (**a**) pH and (**b**) gas pressure over time following test product administration in M-SHIME^®^ short-term colonic incubations. Incubations included the negative control (colonic incubation blank medium), NatureKnit™ (1.667 g fiber/L, 3.333 g/L total), inulin (1.667 g fiber/L), and psyllium (1.667 g fiber/L). Donors A, B, and C represent three individual healthy human fecal donors, and average donor represents the average of the three donors. Incubations were performed in triplicate (*n* = 3) and the results are presented as mean ± standard deviation. Statistical analysis was performed over the course of the entire colonic incubation phase (i.e., between 0 h and 48 h). Paired student’s *t*-tests were used to compare changes observed for the test products versus negative control. A *p*-value of <0.05 was considered statistically significant. Different letters above the bars indicate statistically significant differences between test conditions, while no significant differences were observed between test conditions that share the same letter. M-SHIME^®^ = Mucosal Simulator of the Human Intestinal Microbial Ecosystem.

Changes in pH and gas over time for individual donors and overall (average of all donors) are shown in Figure 1. Test product administration resulted in a significantly greater pH reduction compared with the negative control both at the individual donor level and for the average of all donors (Figure 1a). The strongest overall decrease was observed with NatureKnit™, followed by the purified fibers inulin and psyllium. Additionally, the pH decrease was continuous over the course of the 48 h period with NatureKnit™, while inulin had a sharp pH decrease between 0 h and 6 h, followed by a subsequent increase between 6 h and 24 h.

Overall gas production increased over time for the negative control and with the test product and was significantly greater with the test products versus negative control (Figure 1b). The increase in gas pressure was similar with NatureKnit™ and inulin, and lower for psyllium.

### 3.2. Microbial Metabolic Activity

**Figure 2:**
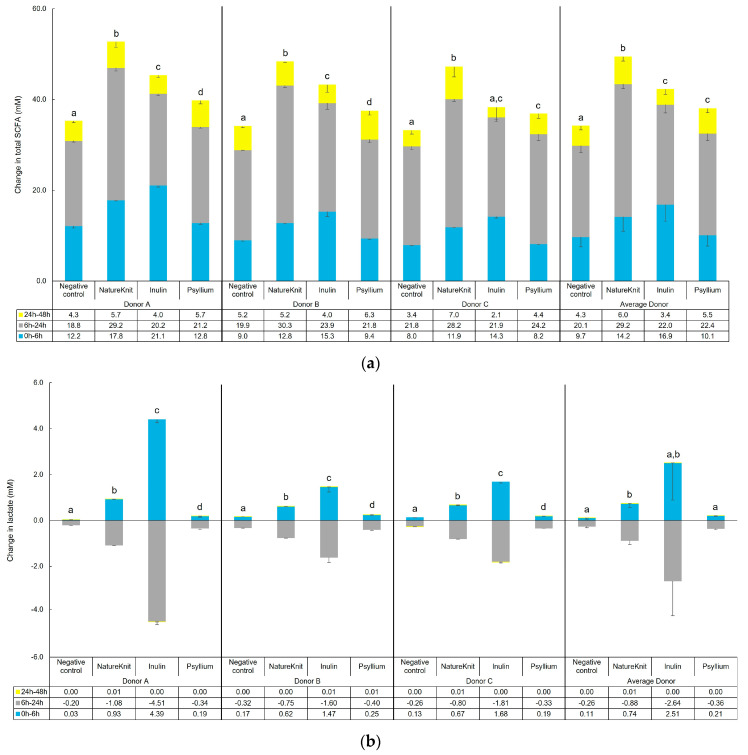

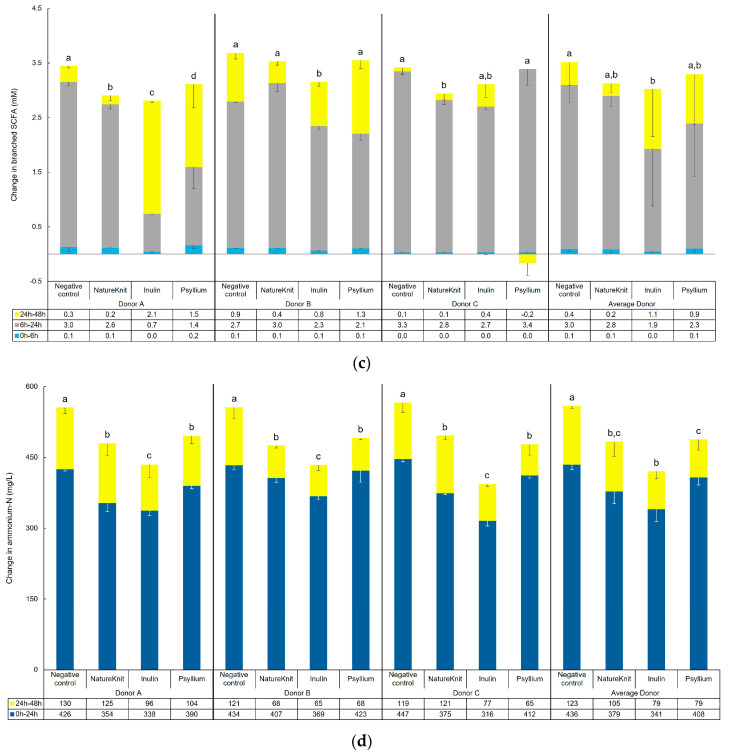
Changes in (**a**) total SCFA, (**b**) lactate, (**c**) branched SCFA, and (**d**) ammonium-N over time following test product administration in M-SHIME^®^ short-term colonic incubations. Incubations included the negative control (colonic incubation blank medium), NatureKnit™ (1.667 g fiber/L, 3.333 g/L total), inulin (1.667 g fiber/L), and psyllium (1.667 g fiber/L). Donors A, B, and C represent three individual healthy human fecal donors, and average donor represents the average of the three donors. Incubations were performed in triplicate (*n* = 3) and the results are presented as mean ± standard deviation. Statistical analysis was performed over the course of the entire colonic incubation phase (i.e., between 0 h and 48 h) or over the course of the initial time interval (i.e., between 0 h and 6 h; lactate only). Paired student’s *t*-tests were used to compare changes observed for the test products versus negative control. A *p*-value of <0.05 was considered statistically significant. Different letters above the bars indicate statistically significant differences between test conditions, while no significant differences were observed between test conditions that share the same letter. M-SHIME^®^ = Mucosal Simulator of the Human Intestinal Microbial Ecosystem; SCFA = short chain fatty acid.

Changes in microbial metabolic activity following test product administration are shown in Figure 2. Changes in total SCFA levels were most pronounced between 6 h and 24 h (Figure 2a). The changes were significantly greater with each of the test products compared to the negative control with all individual donors (except inulin with Donor C) and for the average of all donors (Figure 2a). The greatest change in SCFA levels was observed with NatureKnit™ (change in total SCFA: 0 h to 6 h, +14.2 mM; 6 h to 24 h, +29.2 mM; 24 h to 48 h, +6.0 mM), which was significantly greater (*p* < 0.05) than with either inulin (change in total SCFA: 0 h to 6 h, +16.9 mM; 6 h to 24 h, +22.0 mM; 24 h to 48 h, +3.4 mM) or psyllium (change in total SCFA: 0 h to 6 h, +10.1 mM; 6 h to 24 h, +22.4 mM; 24 h to 48 h, +5.5 mM). The difference in change in total SCFA production between NatureKnit™ and the purified fibers was numerically greatest between 6 h to 24 h. Changes in individual SCFA levels are shown in Supplemental Figure S1. Acetate levels followed a similar pattern as total SCFA, though the difference between inulin and psyllium tended to be more pronounced (Supplemental Figure S1a). Propionate levels were significantly enhanced with each test product versus the negative control and for each individual donor and overall, but the differences between the test products were less pronounced than for total SCFA (Supplemental Figure S1b). Butyrate levels were less affected by the test products compared with the other SCFAs and appeared to be highly donor-dependent; significant differences between the negative control and the test products were not observed for the average of all donors (Supplemental Figure S1c). Overall, NatureKnit™ resulted in greater production of total SCFAs and individual SCFAs as compared to the purified fibers inulin and psyllium.

Lactate levels were increased between 0 h and 6 h and then decreased between 6 h and 24 h with the negative control and test products (Figure 2b). These changes were most pronounced with inulin, followed by NatureKnit™ and psyllium. The changes in lactate levels with inulin administration were donor-dependent, being greatest with Donor A compared with Donors B and C.

For all test conditions, including the negative control, branched SCFA levels had very little increase between 0 h and 6 h; the levels increased greatly between 6 h and 24 h, and further still, though to a lesser extent, between 24 h and 48 h. The overall level of branched SCFA increase tended to be greater with the negative control compared with the test products, though across donors the difference versus the negative control only reached significance with inulin (Figure 2c). For some individual donors, levels were significantly lower versus the negative control for NatureKnit™ (Donors A and C), inulin (Donor A and B), and psyllium (Donor A). For all test conditions, the greatest increase in ammonium was observed between 0 h and 24 h; the level continued to increase but to a lesser extent between 24 h and 48 h (Figure 2d). Overall, ammonium levels were significantly lower for all test products versus the negative control for each individual donor and for the average of all donors. For change in ammonium level, the greatest difference between test product and negative control was observed with inulin.

### 3.3. Microbial Community Composition

**Figure 3:**
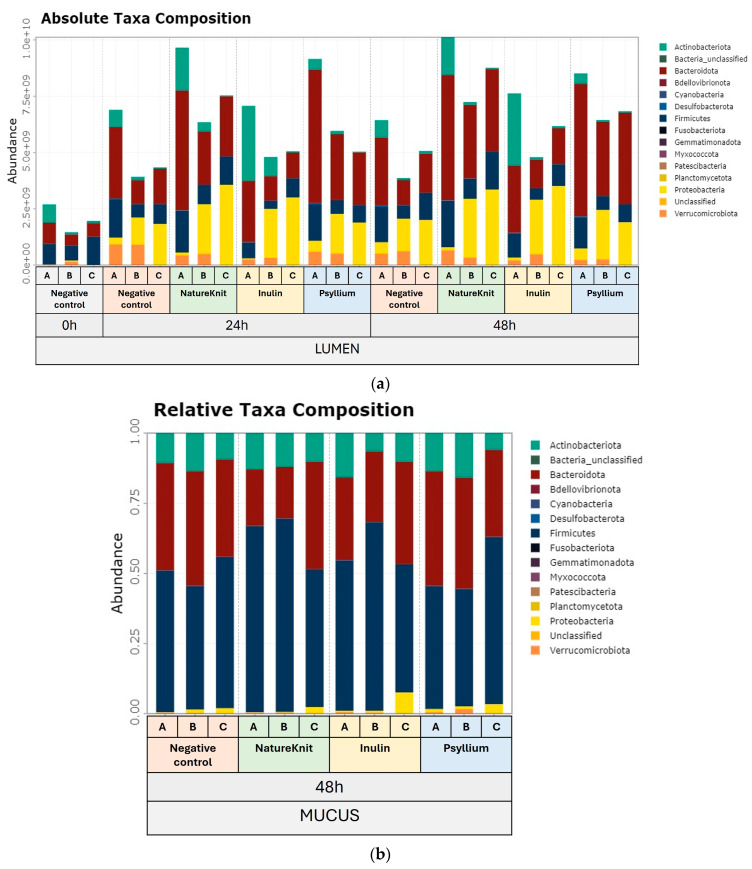
Stacked bar plots showing (**a**) absolute phyla abundances (cells/mL) in the lumen compartment and (**b**) relative phyla abundances in the mucosal compartment. Incubations included the negative control (colonic incubation blank medium), NatureKnit™ (1.667 g fiber/L, 3.333 g/L total), inulin (1.667 g fiber/L), and psyllium (1.667 g fiber/L). Donors A, B, and C represent three individual healthy human fecal donors. Incubations were performed in triplicate (*n* = 3). Flow cytometry was used to determine the total number of bacterial cells in the luminal samples.

The bacterial community composition at the phylum level is shown in Figure 3. In the luminal compartment, the absolute bacterial abundance was increased with all test products relative to the negative control (Figure 3a). For all donors, the strongest effect was observed for NatureKnit™ (+170% on average, *p* = 1 × 10^−8^), followed by psyllium (+142% on average, *p* = 5 × 10^−5^), and inulin (+121% on average, *p* = 1 × 10^−5^). Firmicutes and Bacteroidota were the most abundant phyla, along with Actinobacteriota for Donor A and Proteobacteria for Donors B and C. Assessment of relative abundance in the mucosal compartment revealed that the main phyla for all conditions were Firmicutes, Bacteroidota, and Actinobacteriota (Figure 3b).

**Figure 4:**
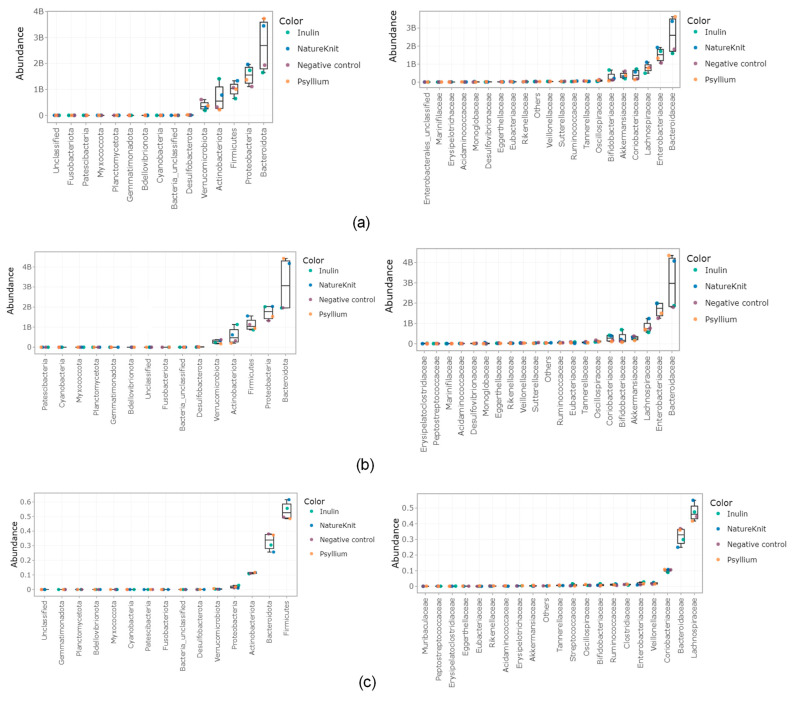
Jitter plots showing (**a**) the absolute abundance (cells/mL) of the top 20 most abundant phyla and families in the lumen compartment after 24 h, (**b**) the absolute abundance (cells/mL) of the top 20 most abundant phyla and families in the lumen compartment after 48 h, and (**c**) the relative abundance of the top 20 most abundant phyla and families in the mucosal compartment after 48 h. Incubations included the negative control (colonic incubation blank medium), NatureKnit™ (1.667 g fiber/L, 3.333 g/L total), inulin (1.667 g fiber/L), and psyllium (1.667 g fiber/L). Incubations were performed for each donor in triplicate (per donor, *n* = 3; total, *n* = 9). Each dot represents the average across donors.

Absolute abundance in the lumen compartment and relative abundance in the mucosal compartment at the phylum and family level are shown in Figure 4. At the 24 h timepoint, the Bacteroidota phylum was enriched in the lumen compartment following the addition of NatureKnit™ and psyllium; the opposite was observed with inulin (Figure 4a). The Bacteroidota enrichment was linked to the *Bacteroidacea* family. The Proteobacteria phylum was upregulated in all conditions and was mainly linked to an enrichment of the *Enterobacteriaceae* family. An enrichment of members of the Firmicutes phylum and the *Lachnospiraceae* family specific to NatureKnit™ was observed. NatureKnit™ and inulin supported an increase in the Actinobacteria phylum that was linked mainly to enrichments of the *Coriobacteriaceae* family in both conditions and the *Bifidobacteriaceae* family with inulin. The findings at 48 h were largely similar to those at 24 h with a few exceptions (Figure 4b). In the mucosal compartment (48 h), the Firmicutes phylum was enriched with NatureKnit™ and inulin, but not with psyllium; this was mainly attributed to an increase in members of the *Lachnospiracea* family (Figure 4c).

**Figure 5:**
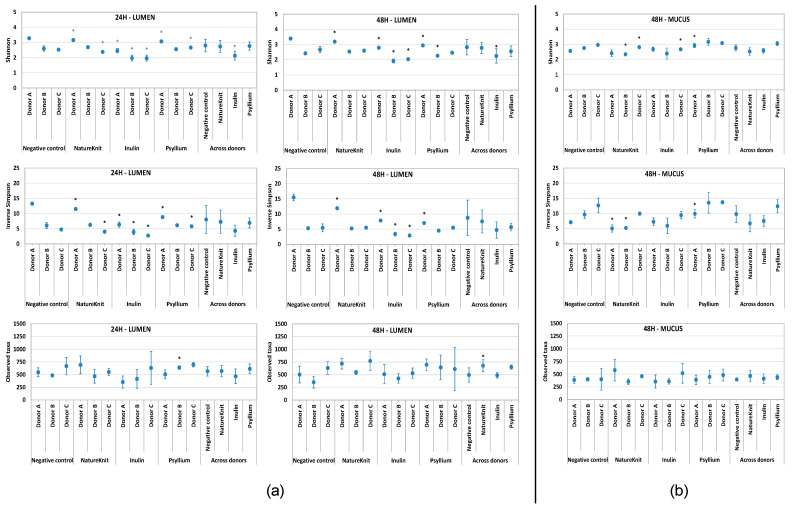
Effect of the test products on alpha diversity as calculated by the observed taxa, Shannon, and inverse Simpson indexes in the (**a**) lumen compartment (24 h and 48 h) and (**b**) mucosal compartment (48 h). Incubations included the negative control (colonic incubation blank medium), NatureKnit™ (1.667 g fiber/L, 3.333 g/L total), inulin (1.667 g fiber/L), and psyllium (1.667 g fiber/L). Donors A, B, and C represent three individual healthy human fecal donors. Incubations were performed in triplicate (*n* = 3). Paired student’s *t*-tests were used to compare each test product with the negative control. A *p*-value of <0.05 was considered statistically significant; the asterisk represents a significant difference versus negative control.

Results for the three alpha diversity indexes are shown in Figure 5. In the lumen compartment, the observed taxa index showed a tendency for increased bacterial richness with NatureKnit™ and psyllium that was significant with NatureKnit™ at 48 h across donors (negative control, 496 observed taxa; NatureKnit™, 680 observed taxa; *p* < 0.05) (Figure 5a). The Shannon and inverse Simpson indices indicated that diversity tended to decrease for all conditions in both colonic compartments after 24 and 48 h (Figure 5a,b). In the lumen compartment, diversity, as measured by the Shannon index, was significantly decreased with inulin relative to the negative control for each individual donor and across all donors at 24 h and 48 h (Figure 5a).

## 4. Discussion

This study used a short-term single-stage colonic M-SHIME^®^ model to investigate changes in microbial metabolism and community composition following administration of NatureKnit™, a proprietary blend of diverse fruit and vegetable fibers rich in naturally occurring bound polyphenols, compared with inulin or psyllium, both purified fibers. All test products were fermented by the colonic microbiota of three different donors as evidenced by changes in pH and gas pressure over time, though the kinetics of these changes differed among the products. Further, SCFA production was significantly increased compared with the negative control for all test products, but this was most notable for NatureKnit™. Measures of microbial community composition demonstrated a shift in the makeup of the microbiota following the addition of the test products. Absolute bacterial abundance increased in the lumen compartment with all test products and was more pronounced with NatureKnit™ than with the purified fibers. The shift in community composition included an increase in the absolute and relative abundances of families capable of producing SCFAs. Additionally, NatureKnit™ tended to support an increase in bacterial richness, with a significantly greater number of unique taxa in the lumen compartment at 48 h compared to the negative control.

The pattern of slower decrease in pH and slower increase in gas production over time with NatureKnit™ compared with the isolated fibers suggests a more gradual fermentation with a proprietary blend of natural fruit and vegetable fibers. Slower fermentation of fibers is generally considered beneficial as it may allow for the delivery of dietary fibers to both the proximal and distal colon [58]. Rapid fermenting fibers are generally fully metabolized in the proximal colon, meaning that the resulting beneficial metabolites, such as SCFAs, are limited to that area of the colon [59]. Undigested proteins are often fermented by proteolytic bacteria in the distal colon. For this reason, metabolites of proteolytic fermentation such as branched SCFAs and ammonia are present at a higher concentration in the distal versus proximal colon [59,60]. The presence of dietary fibers in the distal colon may benefit the host by supporting the growth of saccharolytic bacteria, potentially reducing the activity of proteolytic bacteria [58]. The slower fermentation profile observed upon administration of NatureKnit™ could also in part be related to the gradual release of the bound polyphenols in the colonic environment. Indeed, the fiber-polyphenol bonds can be broken by the action of the gut microbial community, allowing both compounds to exert their potential beneficial effects. Synergistic effects between fibers and polyphenols were previously observed in in vitro studies, but further confirmation based on in vivo trials is still needed [61,62]. Together, this suggests a potential benefit for natural fruit and vegetable fiber blends containing bound polyphenols over purified fibers alone, though further investigation is recommended to more precisely confirm the delivery of undigested fibers to the distal colon and to interrogate the prebiotic role of bound polyphenols.

The overall increase in gas pressure for each test product was within the acceptable range of <100 kPa. Psyllium had the lowest increase in gas pressure, followed by NatureKnit™ and Inulin. The amount of fiber used in this study was well below the amount that would be expected to produce a sufficiently high level of gas pressure to result in discomfort in humans; however, higher doses of purified, rapidly fermenting fibers have been associated with increased bloating and flatulence [63].

SCFA levels were significantly increased relative to the negative control with all test products; the SCFA level was significantly higher with NatureKnit™ than with inulin or psyllium. This, together with the fact that inulin is a well-established prebiotic [27], confirms the prebiotic effects of NatureKnit™ and suggests a potential additional benefit of naturally occurring polyphenols. The increased production of SCFAs may be explained by the changes observed in the microbial community composition. For example, members of the *Bifidobacteriaceae* family are known to produce acetate [64] and the absolute abundance of the *Bifidobacteriaceae* was increased in the lumen compartment, with inulin and NatureKnit™ (to a lesser extent). The absolute abundance of the *Bacteroidaceae* family, containing bacterial species that are able to produce acetate and/or propionate [65], was also enhanced in the lumen environment with NatureKnit™ and psyllium. NatureKnit™ also supported an increase in the abundance of the *Lachnospiraceae* family in the lumen and mucosal environment. Members of this bacterial family are able to produce acetate, propionate, and/or butyrate [66]. While butyrate was not significantly increased relative to the negative control across all donors, it was significantly increased when looking at the levels for each individual donor. Given that SCFAs have several benefits to human health, including anti-diabetes, anti-obesity, anti-inflammatory, anticancer, immunoregulatory, cardioprotective, neuroprotective, and hepatoprotective activities, the increase in SCFAs following the addition of NatureKnit™, inulin, or psyllium suggests the test products may have health benefits for humans [67]. Our findings are in line with previous studies showing that exposure to both inulin and psyllium increases SCFA levels in humans [28,29,68] and newly demonstrates the ability of a fruit and vegetable fiber blend, rich in naturally occurring bound polyphenols, to increase SCFA levels. 

Compared with the negative control, there was a tendency for the test products to reduce the production of branched SCFAs and ammonium, which are byproducts of proteolytic fermentation and are generally considered to have a negative effect on host health. For example, high levels of branched SCFAs are associated with depression and cortisol levels [69] and it has been suggested that branched SCFAs may play a role in regulating glucose and lipid metabolism [70]. Prolonged exposure to branched SCFAs, ammonium, and other protein fermentation products can damage colonic epithelial cells [71]. The reduction in branched SCFA with NatureKnit™ reached significance for two of the three donors but not with the average of all donors, which was similar to the findings for inulin except the latter did reach significance across donors. With all the test products, ammonium was significantly reduced versus the negative control, but the effect was strongest with inulin.

In addition to altering the composition of the microbial community, each of the test products stimulated a significant increase in the absolute abundance of gut bacteria. An increased total bacterial abundance can potentially contribute to improved donor health. For example, differences in fecal microbial load were previously observed between diseased and healthy individuals, with a 100-fold lower fecal microbial load being observed in patients with Crohn’s disease as compared to the healthy control group [72]. There was also a tendency for an increased bacterial richness with NatureKnit™ and psyllium that was significant for NatureKnit™ at 48 h. Decreased bacterial richness is associated with a number of diseases, disorders, and negative health outcomes, as reviewed in [73,74]. 

Although these in vitro findings are encouraging when considering whether NatureKnit™ may have prebiotic effects in humans, we acknowledge that the precise biological response should be elucidated in future in vitro studies and/or human clinical trials. The SHIME^®^ technology platform could, for example, be used to study the long-term effects of NatureKnit™ following repeated intake as it is hypothesized that repeated intake of NatureKnit™ would result in a buildup of the observed short-term effects. Additionally, while many of the observations for NatureKnit™ were consistent across the three donors, some changes, such as the effect on butyrate levels, were donor-specific. Thus, in addition to long-term in vitro or in vivo studies, studies on a larger number of donors are also needed to better understand such donor-specific effects.

## 5. Conclusions

Using a short-term single-stage colonic M-SHIME^®^ model to simulate the human colon, this study demonstrated that NatureKnit™ was well-fermented by the colon microbiota and had several prebiotic effects, including an increase in SCFA production, a shift in the microbial community composition, and an increase in bacterial richness. The prebiotic effects of NatureKnit™ were either similar to or significantly greater than those observed with inulin, one of the most well-established prebiotic fibers. Additionally, the pH and gas pressure results suggest a slower fermentation with NatureKnit™, a blend of natural fruit and vegetable fibers, compared with purified prebiotic fibers. Thus, the prebiotic effect of NatureKnit™ may be more significant and gentler than that of inulin and psyllium. These findings suggest that further investigation into the prebiotic effects of NatureKnit™, including in human studies, is warranted.

## Data Availability

The raw data supporting the conclusions of this article will be made available by the authors on request.

## Supplementary Materials

The following supporting information can be downloaded at: https://www.mdpi.com/article/10.3390/microorganisms13030613/s1, Figure S1: Changes in (a) acetate, (b) propionate, and (c) butyrate over time following test product administration in M-SHIME^®^ short-term colonic incubations.

## Abbreviations

The following abbreviations are used in this manuscript:

M-SHIME^®^	Mucosal Simulator of the Human Intestinal Microbial Ecosystem
SCFA	Short-chain fatty acids
GIT	Gastrointestinal tract
DNA	Deoxyribonucleic acid
rRNA	Ribosomal ribonucleic acid
out	Operational Taxonomic Units
