# Supplementary material for: Effects of NatureKnit™, a Blend of Fruit and Vegetable Fibers Rich in Naturally Occurring Bound Polyphenols, on the Metabolic Activity and Community Composition of the Human Gut Microbiome Using the M-SHIME® Gastrointestinal Model
Source: Microorganisms. 2025 Mar 7;13(3):613. doi: 10.3390/microorganisms13030613 (PMC11944907; doi:10.3390/microorganisms13030613)
Supplement: Supplementary file 1 [file microorganisms-13-00613-s001.zip › microorganisms-3487673-supplementary.pdf]

Supplemental Material

Effects of NatureKnit™, a blend of fruit and vegetable fibers rich in naturally occurring bound polyphenols, on the metabolic activity and community composition of the human gut microbiome using the M-SHIME® gastrointestinal model

Govaert et al.

Figure S1. Changes in (**a**) acetate, (**b**) propionate, and (**c**) butyrate over time following test product administration in M-SHIME® short-term colonic incubations.

Incubations included the negative control (colonic incubation blank medium), NatureKnit™ (1.667 g fiber/L, 3.333 g/L total), inulin (1.667 g fiber/L), and psyllium (1.667 g fiber/L). Donors A, B, and C represent three individual healthy human fecal donors, and average donor represents the average of the three donors. Incubations were performed in triplicate (n = 3) and the results are presented as mean ± standard deviation. Statistical analysis was performed over the entire colonic incubation phase (i.e., between 0 h and 48 h). Paired student's t-tests were used to compare changes observed for the test products versus negative control. A p-value of < 0.05 was considered statistically significant. Different letters above the bars indicate statistically significant differences between test conditions, while no significant differences were observed between test conditions that share the same letter.

M-SHIME® = Mucosal Simulator of the Human Intestinal Microbial Ecosystem.

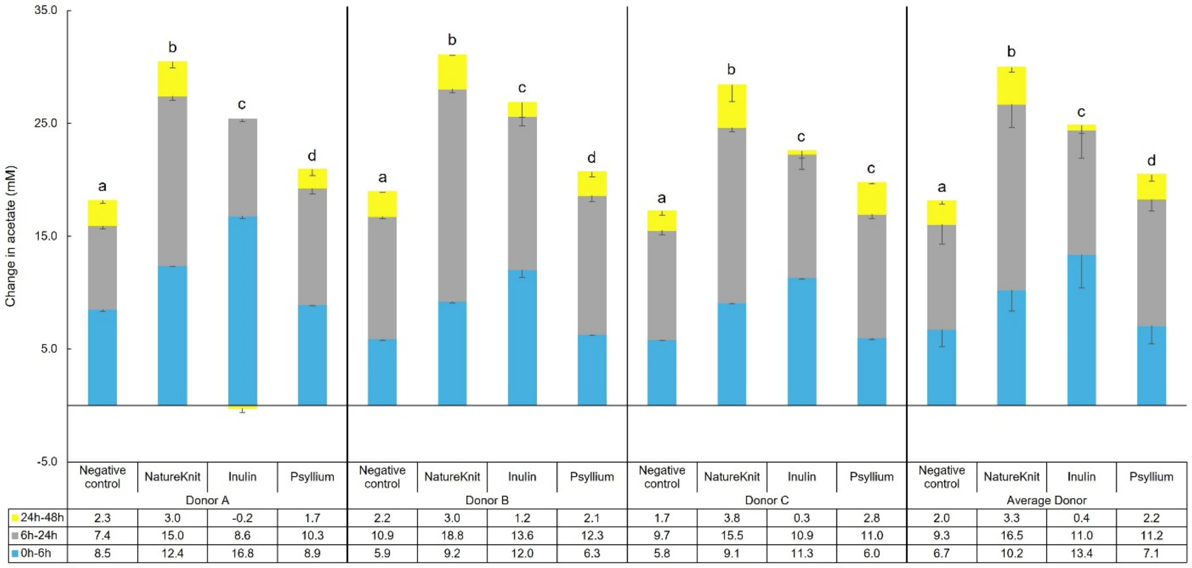

	Donor A Negative control	Donor A NatureKnit	Donor A Inulin	Donor A Psyllium	Donor B Negative control	Donor B NatureKnit	Donor B Inulin	Donor B Psyllium	Donor C Negative control	Donor C NatureKnit	Donor C Inulin	Donor C Psyllium	Average Donor Negative control	Average Donor NatureKnit	Average Donor Inulin	Average Donor Psyllium
24h-48h	2.3	3.0	-0.2	1.7	2.2	3.0	1.2	2.1	1.7	3.8	0.3	2.8	2.0	3.3	0.4	2.2
6h-24h	7.4	15.0	8.6	10.3	10.9	18.8	13.6	12.3	9.7	15.5	10.9	11.0	9.3	16.5	11.0	11.2
0h-6h	8.5	12.4	16.8	8.9	5.9	9.2	12.0	6.3	5.8	9.1	11.3	6.0	6.7	10.2	13.4	7.1

(**a**)

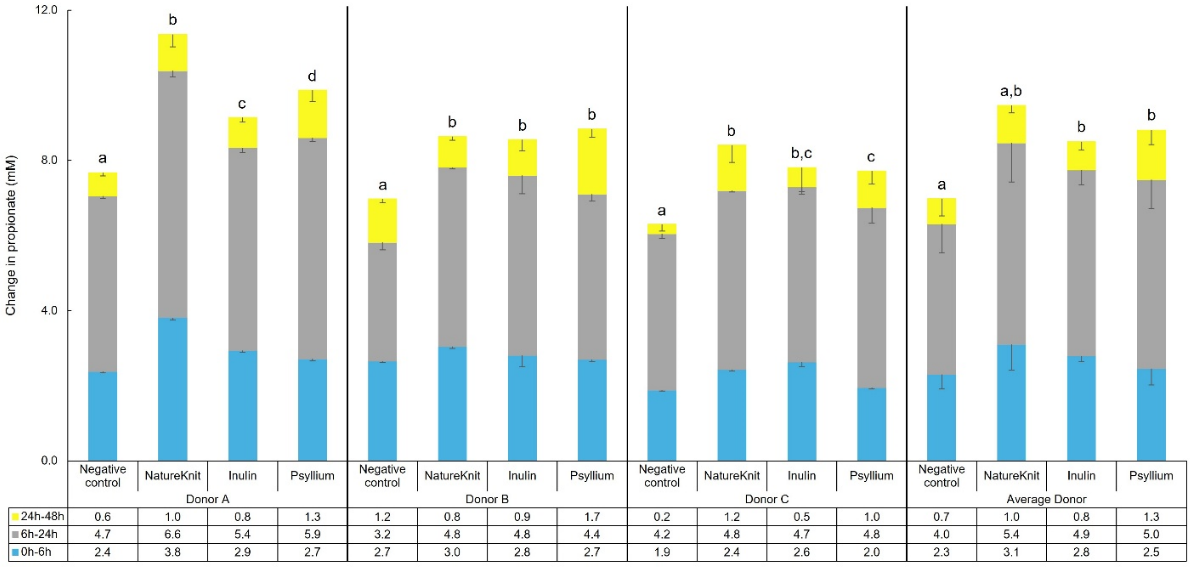

	Negative control	NatureKnit	Inulin	Psyllium	Negative control	NatureKnit	Inulin	Psyllium	Negative control	NatureKnit	Inulin	Psyllium	Negative control	NatureKnit	Inulin	Psyllium
	Donor A				Donor B				Donor C				Average Donor			
24h-48h	0.6	1.0	0.8	1.3	1.2	0.8	0.9	1.7	0.2	1.2	0.5	1.0	0.7	1.0	0.8	1.3
6h-24h	4.7	6.6	5.4	5.9	3.2	4.8	4.8	4.4	4.2	4.8	4.7	4.8	4.0	5.4	4.9	5.0
0h-6h	2.4	3.8	2.9	2.7	2.7	3.0	2.8	2.7	1.9	2.4	2.6	2.0	2.3	3.1	2.8	2.5

(**b**)

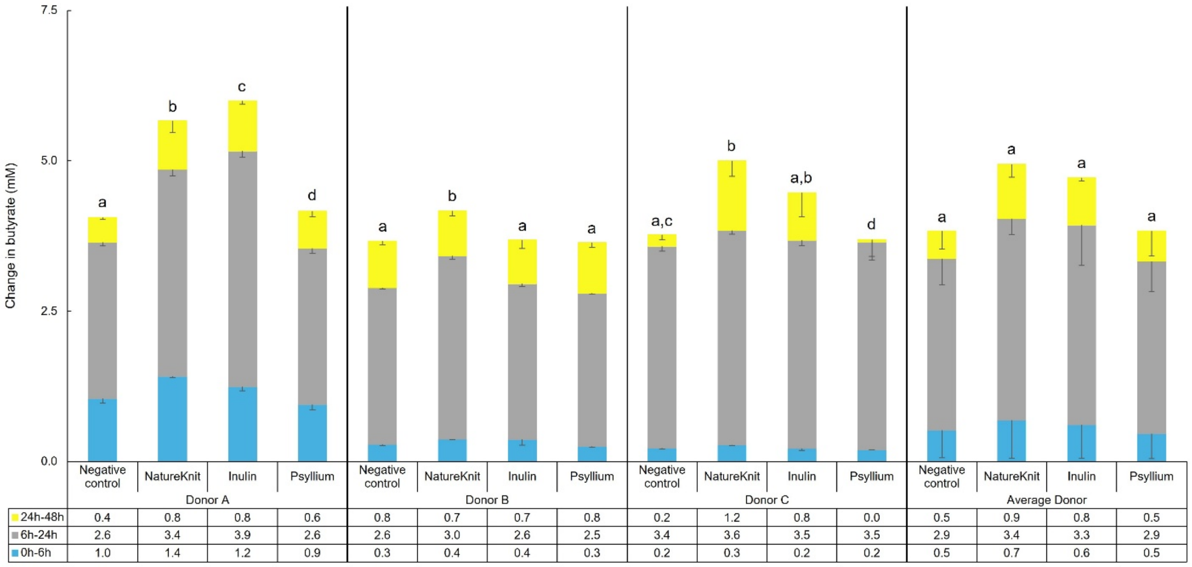

	Negative control	NatureKnit	Inulin	Psyllium	Negative control	NatureKnit	Inulin	Psyllium	Negative control	NatureKnit	Inulin	Psyllium	Negative control	NatureKnit	Inulin	Psyllium
	Donor A				Donor B				Donor C				Average Donor			
24h-48h	0.4	0.8	0.8	0.6	0.8	0.7	0.7	0.8	0.2	1.2	0.8	0.0	0.5	0.9	0.8	0.5
6h-24h	2.6	3.4	3.9	2.6	2.6	3.0	2.6	2.5	3.4	3.6	3.5	3.5	2.9	3.4	3.3	2.9
0h-6h	1.0	1.4	1.2	0.9	0.3	0.4	0.4	0.3	0.2	0.3	0.2	0.2	0.5	0.7	0.6	0.5

(**c**)
